# Spotting senile systemic amyloidosis: why we miss it

**DOI:** 10.1186/1750-1172-10-S1-P51

**Published:** 2015-11-02

**Authors:** Marianna Fontana, Cristina C Quarta, Janet Gilbertson, Thirusha Lane, Carol J Whelan, Julian D Gillmore, Philip N Hawkins, James C Moon

**Affiliations:** 1 National Amyloidosis Centre, UCL, NW3 2QG, London, UK; 2 Barts Heart Centre, Barts Hospital, E2 9JX, London, UK

## Objectives

To analyze the geometric pattern of cardiac hypertrophy in patients with wild-type transthyretin amyloidosis (wtATTR, formerly called senile amyloid) using cardiovascular magnetic resonance (CMR) and to analyze the diagnostic implications.

## Background

wtATTR amyloidosis is an under-diagnosed and underappreciated cause of heart failure. CMR adds value diagnostically for morphological phenotype and tissue characterization.

## Methods

At a national referral centre, over 4 years, 87 consecutive recruited patients wtATTR amyloidosis underwent CMR. The diagnosis was confirmed by histological proof of amyloid (62%), exclusion of TTR mutations (100%) and characteristic features including bone tracer scanning (DPD grade 2-3 in 100%). CMR had precipitated the referrals in 71% of the cohort. Standard long and short axis cines derived the presence and distribution of LVH, relative wall thickness (RTW), inversion of the septal curvature and LV remodeling patterns were determined.

## Results

There were 82 patients with wt ATTR amyloidosis (82 males (94%), age 75 ± 7 years). Patients with wtATTR amyloidosis had increased LV mass index, EF at the lower limit of normal range and markedly reduced indexes of longitudinal function. LV mass was always large compared to cavity size, as expected. However – there was far more asymmetric hypertrophy than expected – 61% of patients had the septum >1.5x thicker than the posterior wall. Inversion of the septal curvature was found in 34% of patients, features typically associated with hypertrophic cardiomyopathy. Tissue characterization with LGE was typical of amyloidosis in 100% of cases (transmural LGE in 73%, subendocardial in 27%).

## Conclusions

CMR is a major source of diagnosis of wtATTR. The majority of patients with wtATTR amyloidosis have a pattern of hypertrophy traditionally thought associated with hypertrophic cardiomyopathy rather than amyloid with asymmetric septal hypertrophy and reverse septal curvature.

